# Microorganisms Detected in Intussusception Cases and Controls in Children <3 Years in South Africa From 2013 to 2017

**DOI:** 10.1093/ofid/ofad458

**Published:** 2023-09-04

**Authors:** Nicola Anne Page, Rembuluwani Netshikweta, Jacqueline E Tate, Shabir A Madhi, Umesh D Parashar, Michelle J Groome, the South African Intussusception Surveillance Group, Marion Arnold, Milind Chitnis, Sharon Cox, Corné de Vos, Mari Kirsten, Susanna M le Grange, Jerome Loveland, Sello Machaea, Ashwini Maharaj, Aletha Withers

**Affiliations:** National Institute for Communicable Diseases, A Division of the National Health Laboratory Services, Johannesburg, South Africa; Department of Medical Virology, Faculty of Health Sciences, University of Pretoria, Pretoria, South Africa; National Institute for Communicable Diseases, A Division of the National Health Laboratory Services, Johannesburg, South Africa; Division of Viral Diseases, National Center for Immunization and Respiratory Diseases, Centers for Disease Control and Prevention, Atlanta, Georgia, USA; South African Medical Research Council Vaccines and Infectious Diseases Analytics Research Unit (VIDA), Faculty of Health Sciences, University of the Witwatersrand, Johannesburg, South Africa; Division of Viral Diseases, National Center for Immunization and Respiratory Diseases, Centers for Disease Control and Prevention, Atlanta, Georgia, USA; South African Medical Research Council Vaccines and Infectious Diseases Analytics Research Unit (VIDA), Faculty of Health Sciences, University of the Witwatersrand, Johannesburg, South Africa

**Keywords:** adenovirus, etiology, intussusception, rotavirus, vaccination

## Abstract

A matched case-control evaluated infectious etiologies in children <3 years in post-rotavirus vaccine intussusception surveillance. Adenovirus and adenovirus types C, A, and B were detected more frequently in cases versus controls at statistically significant values. Wild-type rotavirus, rotavirus vaccine strains, and human herpesvirus were not associated with intussusception.

Intussusception (ISS), a rare cause of bowel obstruction, occurs naturally with rates varying considerably and children in Africa presenting later for treatment, leading to complications and death [1]. Although the exact trigger is unknown, human adenovirus (HAdV), HAdV type C (HAdV-C), human herpesvirus 6 (HHV-6), and rotavirus vaccines have been associated with ISS [1]. Nonpharmaceutical interventions after the severe acute respiratory syndrome coronavirus 2 pandemic saw a reduction in ISS cases in South Korea [2, 3]. In one study, ISS rates declined from 16.97–13.94 per 100 000 population (prepandemic years 2017–2019) to 5.98 in 2020 [2]. In another, ISS cases decreased temporally with a decline in communicable diseases [3]. These results strengthen the hypothesis that a portion of ISS cases may have an infectious etiology or trigger.

Rotavirus vaccines have been associated with ISS in some settings. Intussusception surveillance was established in South Africa (SA) after monovalent rotavirus vaccine (RV1) introduction in 2009. The ISS risk 21 days after RV1 administration was not higher than background risk in South African infants [4]. However, delayed presentation led to poorer outcomes and a 1% ISS-associated mortality [5]. To evaluate potential infectious etiologies of ISS in South African children, stool specimens collected during ISS surveillance from cases and controls were screened for ISS-associated microorganisms.

## METHODS

From 2013 to 2017, children <3 years were recruited from 8 hospitals in 5 provinces [4]. Intussusception cases fulfilled level-1 Brighton Collaboration criteria, whereas controls included children hospitalized for non-ISS surgery and matched by age (date of birth ± 90 days), date of admission (±90 days), and hospital (South African National Clinical Trial Register DOH-27-0913-4183). After enrollment and informed consent, stools were collected within 48 hours of admission [4]. Nucleic acids, extracted from stools, were screened on customized TaqMan array cards ([TAC] Supplementary Table 1) [6]. The pathogens included on the card had either a documented or hypothesized association with intussusception in the published literature. Adenovirus-positive specimens (Adenovirus pan) negative or inconclusive for type C and F targets on TAC were rescreened using HAdV species-specific assays [7]. All specimens were screened for rotavirus using ProsPect Rotavirus Microplate Assay (EIA; Oxoid Ltd., Ely, United Kingdom), and a subset (138 cases and 128 controls) was assessed using conventional polymerase chain reaction (PCR) assays. Rotavirus-positive specimens were genotyped and any G1 strains were sequenced.

Descriptive analyses are presented as medians with interquartile ranges (IQRs) and percentages of microorganism in cases and controls. Microorganism detection was compared in cases and controls using conditional logistic regression to estimate odds ratios (ORs) and 95% confidence intervals (CIs). Where no microorganisms were detected in a specific group, Fishers exact tests were used. All analyses were performed using STATA version 12 with results considered significant at *P* < .05.

### Patient Consent Statement

The patient's written consent was obtained for the study. The design of the work has been approved by the ethics committees of University of the Witwatersrand, University of KwaZulu-Natal, University of Cape Town, University of Stellenbosch, University of the Free State, University of Pretoria, and Walter Sisulu University and registered with the South African National Clinical Trial Register.

## RESULTS

A total of 397 stool specimens from ISS cases and 235 stool specimens from controls were available for testing on TAC (Supplementary Figure 1). Correctly matched controls (n = 170) were available for 170 ISS cases and were included in analysis. In the matched cohort, the median age of cases and controls was 6 months (IQR = 4–8 months and IQR = 5–9, respectively) with 94% (160 of 170) of cases and 96% (163 of 170) of controls receiving at least 1 dose of RV1.

The median number of microorganisms detected was 2 (IQR, 1–4) with no significant difference between cases and controls (ISS cases, 3 pathogens [IQR, 1–4]; controls, 2 pathogens [IQR, 1–4]; *P* = .484). The most frequent microorganisms detected were HAdV (45.3%, 154 of 340 for any detection; 40.6%, 138 of 340 for adenovirus pan), *Clostridioides difficile* (22.1%, 75 of 340), enterovirus (21.2%, 72 of 340), any rotavirus (detection by TAC, EIA, and conventional PCR assays; 12.3%, 42 of 340), enteropathogenic *Escherichia coli* (11.8%, 40 of 340), and norovirus (11.2%, 38 of 340) (Table 1). Intussusception cases were 3 times more likely to have HAdV detected than controls (any detection, OR = 3.18 [95% CI, 1.97–5.14; *P* < .001]; adenovirus pan detection, OR = 3.53 [95% CI, 2.12–5.87; *P* < .001]) (Table 1). The difference was statistically significant at quantification (quantification of cycle [Cq]) values <30 (data not shown) (Table 1). Most HAdV detected were typed (98.7%, 152 of 154) with type C predominant (62%, 95 of 154), and other types were detected at lower levels (type B 10%, type A 9%, type F 9%, type D 2%, type E 1%, and mixed HAdV 6%).

**Table 1.** Comparison of Microorganisms Detected in the Stools of ISS Cases and Matched Controls

Pathogen	Cases (n = 170) n (%)	Controls (n = 170)^a^ n (%)	OR (95% CI)^b^	*P* Value
Adenovirus (any^c^)	101 (59.4)	53 (31.2)	**3.18 (1.97–5.14)**	**<**.**001**
Adenovirus pan^d^ (any Cq value)	93 (54.7)	45 (26.5)	**3.53 (2.12–5.87)**	**<**.**001**
Adenovirus C (any)	65 (38.2)	34 (20.0)	**2.55 (1.52–4.28)**	**<**.**001**
Adenovirus C TAC (any Cq value)	50 (29.2)	24 (14.2)	**2.86 (1.55–5.25)**	.**001**
Adenovirus F (any)	9 (5.3)	10 (5.9)	0.90 (.37–2.21)	.819
Adenovirus A	13 (7.7)	3 (1.8)	**4.33 (1.23–15.2)**	.**022**
Adenovirus B	14 (8.2)	5 (2.9)	**2.80** (**1.01–7.77)**	.**048**
Adenovirus D	4 (2.4)	0 (0.0)	…	.123^e^
*Clostridioides difficile*	22 (12.9)	53 (31.2)	**0.33 (.18–.58)**	**<**.**001**
Enterovirus	24 (14.1)	48 (28.2)	**0.41 (.24–.73)**	.**002**
EPEC (bfpA/eae)	12 (7.1)	28 (16.5)	**0.30 (.13–.71)**	.**006**
*Helicobacter pylori*	0 (0)	6 (3.5)	…	.030^e^
HHV-6	14 (8.2)	7 (4.1)	2.00 (.81–4.96)	.134
Norovirus GII	11 (6.5)	27 (15.9)	**0.30 (.13–.71)**	.**006**
RotaRix (any)	7 (4.1)	9 (5.3)	0.78 (.29–2.09)	.618
RotaTeq	0 (0)	1 (0.6)	…	1.000^e^
Rotavirus (any)	20 (11.7)	22 (12.9)	0.89 (.47–1.72)	.739

Abbreviations: CI, confidence interval; Cq, quantification of cycle; EPEC, enteropathogenic *Escherichia coli*; HHV-6, human herpesvirus 6; ISS, intussusception; OR, odds ratio; TAC, TaqMan array cards.

NOTE: Statistically significant values highlighted in bold.

a Controls were matched to cases by hospital, date of birth (±90 days), and date of admission (±90 days).

b Odds ratio is determined by conditional logistic regression.

c Any detection of an adenovirus target on the TAC including pan, type C or type F.

d Adenovirus pan assay targets all viral types (A–G).

e Where no pathogens were detected, Fisher’s exact tests were used.

Adenovirus type C was almost 3 times more likely to be detected in cases compared to controls (any detection, OR = 2.55 [95% CI, 1.52–4.28; *P* < .001]; TAC detection, OR = 2.86 [95% CI, 1.55–5.25; *P* = .001]). Adenovirus type A (OR = 4.33 [95% CI, 1.23–15.2; *P* = .022]) and HAdV-B (OR = 2.80 [95% CI, 1.01–7.77; *P* = .048]) were detected more frequently in cases than controls (Table 1). Enteric HAdV-F were not detected at increased levels in ISS cases (5.3% in cases vs 5.9% controls; OR = 0.90 [95% CI, .37–2.21; *P* = .819]) (Table 1). There was no significant difference in the detection of wild-type rotavirus, RotaRix, RotaTeq, and HHV-6 in cases and controls (Table 1).

## DISCUSSION

Our results were similar to an Asian study, utilizing a comprehensive TAC to screen ISS cases and matched controls for associated microorganisms [8]. Adenovirus type C was associated with ISS at any Cq value with the strongest association occurring at Cq values <20 (data not shown). Adenovirus type C persists in the T lymphocytes of tonsils and adenoids, particularly in younger children, and primary or reactivated infections could trigger an ISS event [9]. Furthermore, HAdV were detected in resected, enlarged lymph nodes in 4 patients undergoing ISS surgery, supporting the role of HAdV in ISS [10]. Adenovirus type C (and type B) has been associated with disseminated infections, indicating the ability to move from the tonsils or adenoids to other parts of the body [11]. However, basic science studies investigating the triggers and mechanisms of idiopathic ISS pathogenesis in young children are required.

In addition to HAdV-C, the adenovirus pan target was also detected more frequently in ISS cases than controls. Although enteric HAdV-F was not associated with ISS, HAdV-A and HAdV-B were associated with ISS cases. The Asian study showed an association between non-type C HAdV strains and ISS with a stronger association in Vietnam; however, the HAdV strains were not typed further [8]. A recent literature review showed that HAdV-A had been evaluated in 3 studies with no positive associations and HAdV-B had been evaluated in 4 studies with 1 showing a positive association [12]. Typing of HAdV strains detected in ISS cases is recommended.

Human herpesvirus 6 was detected in more ISS cases than controls in the Asian study [8] as well as a study in Finland [13]. However, no statistically significant difference in HHV-6 detection was noted in cases compared to controls in SA. This may be partly due to the failure to collect and screen blood or tissue specimens because the Finnish study detected HHV-6 in 44% of whole blood specimens and 50% of resected mesenteric lymph nodes [13]. The Finnish study proposed that the combination of HHV-6 infection in intestinal lymph nodes with an intestinal adenovirus infection might trigger intussusception [13], and inclusion of blood and resected specimen in future ISS studies is recommended.

Wild-type rotavirus and rotavirus vaccines were not associated with ISS cases. These findings support the postvaccine introduction ISS surveillance results, which reported no increased risk of ISS in the 21 days after the first and second rotavirus vaccine dose compared to background [4].

The study had several limitations. Only 340 patients (170 cases and 170 controls) were correctly matched, had sufficient stool available for testing, and were included in the analysis. Although 5 of 9 provinces were represented, cases and controls were not enrolled in equal numbers at each site, with 1 site enrolling 45% of participants, which may have biased the results. Although our results were similar to those from Asia and participants were drawn from diverse socioeconomic areas [4], the results may not be generalizable to other study populations. Stools were collected after hospitalization and may not have captured infections occurring before hospitalization. No blood or tissue specimens were collected from cases or controls and, therefore, HHV-6 detection may have been reduced.

## CONCLUSIONS

In conclusion, HAdV was associated with ISS cases, with HAdV-C, HAdV-A, and HAdV-B detected more frequently in cases than controls in SA. Detection of HHV-6, wild-type rotavirus, and rotavirus vaccine strains was not associated with ISS in this study. Continued research on microorganisms associated with ISS and mechanisms of pathogenesis should be considered.

## Supplementary Material

ofad458_Supplementary_Data

## Supplementary Data

Supplementary materials are available at *Open Forum Infectious Diseases* online. Consisting of data provided by the authors to benefit the reader, the posted materials are not copyedited and are the sole responsibility of the authors, so questions or comments should be addressed to the corresponding author.
