# Supplementary material for: Microorganisms Detected in Intussusception Cases and Controls in Children <3 Years in South Africa From 2013 to 2017
Source: Open Forum Infect Dis. 2023 Sep 4;10(9):ofad458. doi: 10.1093/ofid/ofad458 (PMC10500044; doi:10.1093/ofid/ofad458)
Supplement: ofad458_Supplementary_Data [file ofad458_supplementary_data.zip › Supplementary figure 1_ISS_BR.docx]

Surgical controls matched by age, hospital and admission date (n=170)

Surgical controls screened (n=289)

14 – Insufficient stool for screening

Surgical controls enrolled (n=303)

Intussusception cases matched by age, hospital and admission date (n=170)

Intussusception cases screened (n=397)

13 – Insufficient stool for screening

64 – No specimen collected

Intussusception cases enrolled (n=474)

Supplementary figure 1. Patients enrolled with intussusception (level 1 Brighton Collaboration criteria) as cases and for other surgery (including inguinal hernia, anorectal malformations, Hirschsprung’s Disease and umbilical hernia) as controls with numbers of participants used for analysis.
