# Supplementary material for: Microorganisms Detected in Intussusception Cases and Controls in Children <3 Years in South Africa From 2013 to 2017
Source: Open Forum Infect Dis. 2023 Sep 4;10(9):ofad458. doi: 10.1093/ofid/ofad458 (PMC10500044; doi:10.1093/ofid/ofad458)
Supplement: ofad458_Supplementary_Data [file ofad458_supplementary_data.zip › Supplementary table 1_ISS_BR.docx]

Supplementary table 1. Gene targets for pathogens on the Taqman array card for intussusception screening [1].

| Port | |
| --- | --- |
| Left | Right |
| Adenovirus pan (Hexon gene) | Adenovirus F (Fiber gene) |
| Adenovirus C (Hexon gene) | Adenovirus C (Hexon gene) |
| Astrovirus (Capsid gene) | Enterovirus (5’ UTR) |
| Cytomegalovirus (Glycoprotein B) | Cytomegalovirus (Immediate early 2) |
| Epstein-Barr virus (DNA polymerase) | Epstein-Barr virus (DNA polymerase) |
| Human Herpesvirus 6 (Immediate-early gene) | Human Herpesvirus 7 (Structural phosphoprotein) |
| Norovirus genogroup I (ORF1-2) | Norovirus genogroup II (ORF1-2) |
| Rotavirus (NSP3) | Rotavirus (NSP3) |
| Rotarix-specific (NSP2) | Rotateq-specific (VP6) |
| Sapovirus (RdRp) | Clostridioides difficile (tcdA, tcbB) |
| MS2 (RNA extraction control; *MS2g1*) | MS2 (RNA extraction control; *MS2g1*) |
| *Campylobacter jejuni*/*coli* (cadF) | *Campylobacter* pan (Cpn60) |
| *Helicobacter pylori* (ureC) | *Salmonella enterica* (ttr) |
| 18S (Manufacturer positive control) | *Shigella*/enteroinvasive *Escherichia coli* (*ipaH*) |
| *Yersinia enterocolitica* (*lytA*) | Enteroaggregative *Escherichia coli* (aggR) |
| Enteropathogenic *Escherichia coli* (eae) | Enteropathogenic *Escherichia coli* (bfpA) |
| Enterotoxigenic *Escherichia coli* (STh, STp) | Enterotoxigenic *Escherichia coli* (LT) |
| Shiga toxin-producing *Escherichia coli* (*stx1*) | Shiga toxin-producing *Escherichia coli* (*stx2*) |
| *Escherichia coli* O157 (*rfbE*) | *Giardia* spp. (18S rRNA) |
| Phocine Herpesvirus (DNA extraction control; *gB*) | Phocine Herpesvirus (DNA extraction control; *gB*) |
| *Cryptosporidium* spp. (18S rRNA) | *Entamoeba histolytica* (18SrRNA) |
| *Ancyclostoma duodenale* (*ITS2*) | *Necator americanus* (*ITS2*) |
| *Ascaris lumbricoides* (*ITS1*) | *Strongyloides stercoralis* (Dispersed repetitive sequence) |
| Bacterial 16S (PCR control) | *Trichuris trichiura* (18SrRNA) |

[1] Liu J, Gratz J, Amour C, et al. Optimization of Quantitative PCR Methods for Enteropathogen Detection. PLoS One 2016; 11:e0158199.
